# Tuning the Electronic Bandgap of Penta-Graphene from Insulator to Metal Through Functionalization: A First-Principles Calculation

**DOI:** 10.3390/nano14211751

**Published:** 2024-10-31

**Authors:** J. O. Morales-Ferreiro, Gerardo Silva-Oelker, Chandra Kumar, Carlos Zambra, Zeyu Liu, Donovan E. Diaz-Droguett, Diego Celentano

**Affiliations:** 1 Escuela de Ingeniería, Facultad de Ciencias, Ingeniería y Tecnología, Universidad Mayor, Santiago 7500994, Chile; gerardo.silvao@umayor.cl (G.S.-O.); chandra.kumar@umayor.cl (C.K.); 2 Centro de Nanotecnología Aplicada (CNAP), Facultad de Ciencias, Ingeniería y Tecnología, Universidad Mayor, Santiago 7500994, Chile; 3 Facultad de Ingeniería, Universidad de Talca, Camino los Niches Km 1, Curicó 3344158, Chile; czambra@utalca.cl; 4 Department of Applied Physics, School of Physics and Electronics, Hunan University, Changsha 410082, China; liuzyspe@hnu.edu.cn; 5 Instituto de Física, Facultad de Física, Pontificia Universidad Católica de Chile, Casilla 306, Av. Vicuña Mackenna 4860, Macul, Santiago 7820436, Chile; ddiazr@uc.cl; 6 Centro de Investigación en Nanotecnología y Materiales Avanzados (CIEN-UC), Pontificia Universidad Católica de Chile, Av. Vicuña Mackenna 4860, Macul, Santiago 7820436, Chile; dcelentano@uc.cl; 7 Centro de Energía UC, Pontificia Universidad Católica de Chile, Av. Vicuña Mackenna 4860, Macul, Santiago 7820436, Chile; 8 Departamento de Ingeniería Mecánica y Metalúrgica, Pontificia Universidad Católica de Chile, Av. Vicuña Mackenna 4860, Macul, Santiago 7820436, Chile

**Keywords:** penta-graphene, density functional theory, hydrogenated, fluorinated, chlorinated, bandgap, electronic structure

## Abstract

We performed first-principles density functional theory (DFT) calculations to numerically investigate the electronic band structures of penta-graphene (PG), a novel two-dimensional carbon material with a pentagonal lattice structure, and its chemically functionalized forms. Specifically, we studied hydrogenated PG (h-PG), fluorinated PG (f-PG), and chlorinated PG (Cl-PG). We used the generalized gradient approximation (GGA) and the hybrid Heyd–Scuseria–Ernzerhof (HSE06) exchange-correlation functional in the DFT-based software VASP to capture electronic properties accurately. Our results indicate that hydrogenation and fluorination increased the indirect bandgap of PG from 3.05 eV to 4.97 eV and 4.81 eV, respectively, thereby effectively transforming PG from a semiconductor to an insulator. In contrast, we found that chlorination closed the bandgap, thus indicating the metallic behavior of Cl-PG. These results highlight the feasibility of tuning the electronic properties of PG through functionalization, offering insight into designing new materials for nanoelectronic applications.

## 1. Introduction

Two-dimensional (2D) nanomaterials possess compelling properties, versatility, and a wide range of potential applications, making them subjects of interest to scientists and engineers [1]. These nanostructures represent an advanced class of materials with atomic-level thickness, consisting of one to a few layers of atoms [2,3,4,5,6]. Research on 2D materials has rapidly advanced in the last decade; since the successful synthesis of graphene in 2004, there has been considerable interest in exploring novel carbon-based two-dimensional materials. The discovery of graphene—a single layer of carbon atoms densely packed into a two-dimensional honeycomb crystal lattice—has attracted scientific interest due to its physicochemical properties, such as electrical conductivity and surface topography. As a zero-bandgap semimetal, graphene is considered a remarkable material because of its exceptional structural, mechanical, optical, electrical, and thermal properties [7,8,9,10,11]. This zero-bandgap semimetal has potential applications in solar cells [12], field-effect transistors [13,14,15,16], optoelectronics [17], lithium-ion batteries [18], and molecular sensing [19]. Notably, the investigation of new graphene allotropes is of particular interest for modern electronics due to their ability to tune electrical or mechanical properties [20].

Though relevant for current optoelectronics devices, the zero-bandgap characteristic of graphene [21] limits its use in applications such as absorbing layers in solar cells and other nanoelectronic devices. Bandgap-opening strategies, such as strain engineering, oxide reduction, and functionalization, have been developed to address this challenge. Several 2D materials have also been explored as alternatives to graphene. Silicene, an allotrope of silicon, has attracted attention due to its tunable bandgap and high electron mobility. Moreover, other 2D materials, such as phosphorene, blue phosphorene, and boron nitride (h-BN), have been successfully synthesized, and their air stability has been examined; stable materials maintain their properties over time, making them more reliable for practical applications and more accessible to integrate into technologies [22]. Additionally, over the last decade, researchers have focused on various 2D structures like hydroxides, transition metal dichalcogenides (TMDs), MXenes, and hexagonal h-BN to handle their properties. For instance, tuning the energy gap of 2D graphene, silicene, and germanene through modifying structures or doping has been reported [23]. These advancements showcase the various 2D materials explored to overcome graphene’s limitations and tailor properties for specific applications.

Furthermore, 2D materials with a pentagonal-like structure show a unique atomic arrangement, introducing a new category of 2D structures. Two-dimensional pentagonal nanostructures represent a new class of atomic configurations that promise to advance the study of 2D structures. Carbon allotropes, particularly graphene and its derivatives, such as penta-graphene (PG), are known to be stable due to their structural characteristics. In particular, the buckled structure of PG enhances its stability due to the combined sp^2^ and sp^3^ hybridization [23]. Additionally, the PG fully bonded natural structure prevents interaction with oxidizing agents.

Experimentally, PG may be obtained by exfoliating the T12-carbon phase. In particular, Zhang et al. [23] theoretically demonstrated that PG can be synthesized by a chemical exfoliation procedure in T12-carbon. They also reported that PG is metastable, able to withstand temperatures up to 1000 K, and is a wide bandgap semiconductor with an indirect bandgap of 3.25 eV. However, despite PG’s promising properties, this high-energy gap value suggests the requirement for gap-tuning strategies to tailor this 2D structure for optoelectronic applications. Therefore, thorough studies are required to understand the bandgap modulation of PG. In this work, we shed light on the bandgap tuning of PG by numerically studying and comparing the band structure of a series of single-layer 2D carbon-based pentagonal materials, including hydrogenated PG (h-PG), fluorinated PG (f-PG), and chlorinated PG (Cl-PG). The goal was to use first-principles density functional theory [24,25,26] (DFT) to understand how functionalization tunes PG electronic properties by introducing hydrogen, fluorine, and chlorine atoms on both sides of the sheet. Our results reveal that surface modification effectively tuned the electronic properties of PG from semiconducting to insulating in the case of h-PG and f-PG; chlorination, on the other hand, resulted in a semiconductor–metal transition.

This paper is organized as follows. Section 2 describes computational details. Section 3 includes results and a discussion of the band structure and density of states obtained from the calculations. Finally, Section 4 outlines the main conclusions of this work.

## 2. Computational Details

As the Introduction mentions, first-principles DFT calculations were used to calculate electronic properties. First, these pentagonal materials were fully optimized to eliminate non-zero Hellmann–Feynman forces. Next, the band structure and density of states (DOS) were calculated using the DFT programs in the Vienna Ab initio Simulation Package (VASP) [27]. For all DFT calculations in VASP, projector augmented wave (PAW) methods were used to capture interactions between ions and valence electron wavefunctions. The generalized gradient approximation (GGA) parameterized by Perdew, Burke, and Ernzerhof (PBE) was used for the exchange-correlation functional. We also conducted high-accuracy electronic structure calculations using the HSE06 hybrid exchange-correlation functional [28,29,30,31]. Plane waves with an energy cutoff of 500 eV were employed for all calculations. A Monkhorst–Pack k-mesh of 8 × 8 × 1 was used to sample the first Brillouin zone. For the electronic band structure calculation, a finer mesh of 25 × 25 × 1 was used. For monolayer simulations, a large vacuum space of at least 15 Å was left in the z-direction to prevent interactions between the layer and its periodic images in the cross-plane direction (see Table 1).

## 3. Results and Discussion

### 3.1. Optimized Structures

We determined the optimal structures using DFT calculations; the resulting lattice parameters and their comparison to values reported in the literature are presented in Table 2.

**Figure 1:**
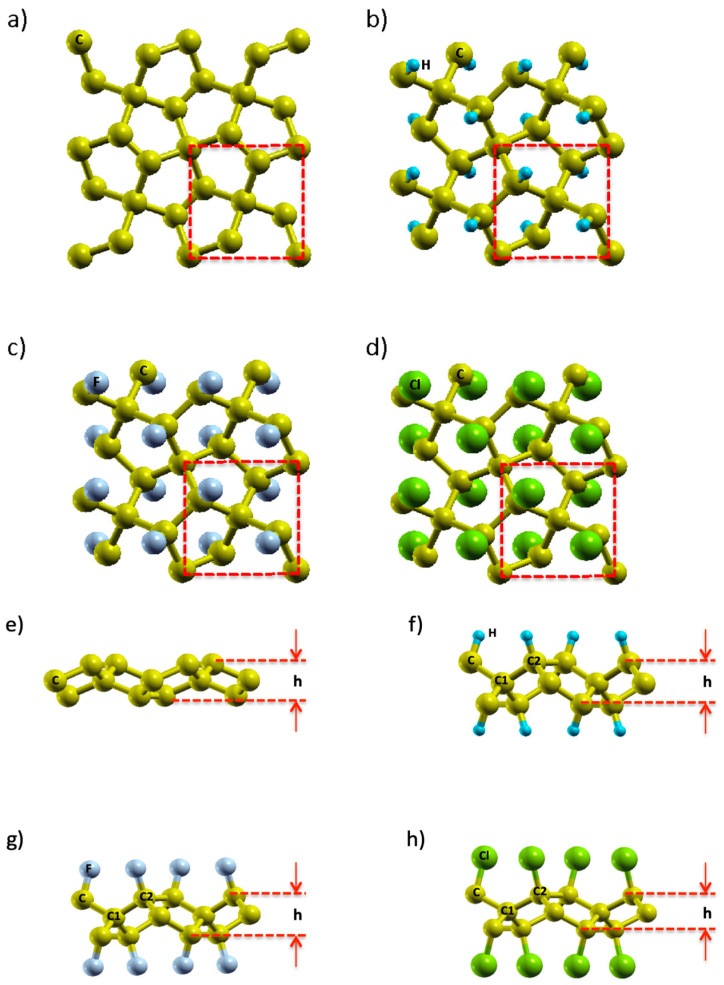
Top views of the optimized crystal structures of (**a**,**e**) PG, (**b**,**f**) h-PG, (**c**,**g**) f-PG, and (**d**,**h**) Cl-PG. The square red dashed lines denote unit cells.

It was verified that structures of PG and functionalized PG simulated in this work all share a tetragonal crystal structure with P-421m symmetry (#113), indicating that the functionalization of h-PG, f-PG, and Cl-PG does not alter the crystallographic symmetry of PG. Top views of the optimized crystal structures by DFT calculation are shown in Figure 1a–d. Each unit cell of PG and functionalized PG consists of a layered crystal structure containing six atoms (two sp^3^- and four sp^2^-hybridized carbon atoms), which are symmetrically shared in PG—the unit cells of PG and functionalized PG are represented with a dashed line.

Moreover, PG, h-PG, f-PG, and Cl-PG bond lengths were calculated. The four-fold and three-fold coordinated carbon atoms of pristine PG were grouped as C_1_ and C_2_, respectively. We found that the bond lengths of C_1_–C_2_ and C_2_–C_2_ were 1.551/1.565/1.668 Å, and 1.55/1.559/1.663 Å for h-PG, f-PG, and Cl-PG, respectively. The bond angles $\theta_{C_{1}–C_{2}–C_{1}}$ and $\theta_{C_{2}–C_{1}–C_{2}}$ were 105.9°/107.2°/114.6° and 116.9°/119.5°/129.7° for h-PG, f-PG, and Cl-PG, respectively. These values suggest the distorted sp^3^ hybridization of the carbon atoms [37].

Figure 1e–h shows the carbon C_1_, C_2_, and the buckling height, *h*. This height is the difference between the top and bottom carbon layers of h-PG, f-PG, and Cl-PG. The buckling height of h-PG, f-PG, and Cl-PG increased to 1.62/1.61/1.55 Å, respectively, from the value 1.21 Å of PG. The optimized parameters of bond lengths, angles, and bandgaps of h-PG, f-PG, and Cl-PG are listed in Table 3.

### 3.2. Electronic Properties

**Figure 2:**
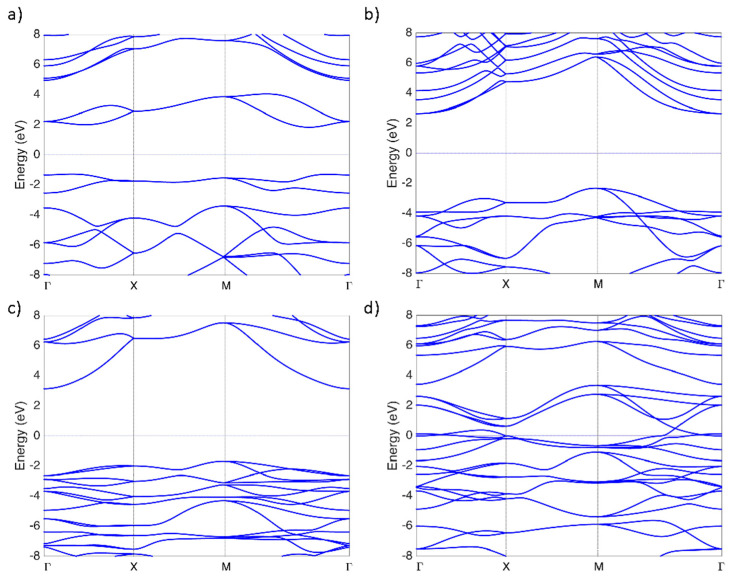
Band structures of (**a**) PG, (**b**) h-PG, (**c**) f-PG, and (**d**) Cl-PG.

**Figure 3:**
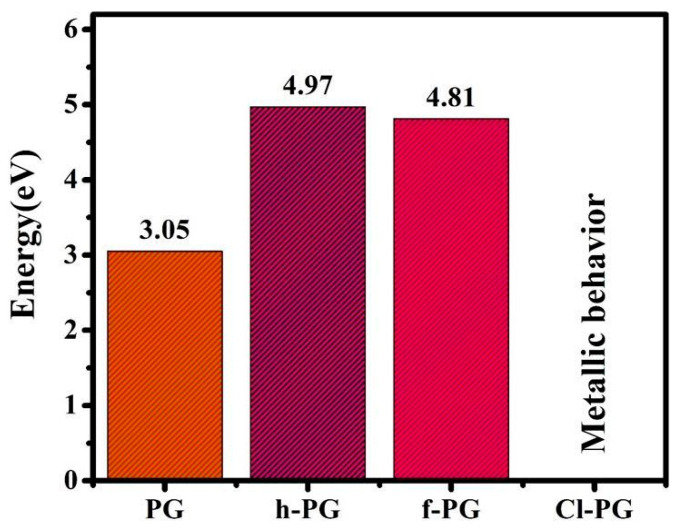
Variation in energy gaps of PG, h-PG, f-PG, and Cl-PG.

As chemical functionalization has been reported to have the possibility of tuning the electronic behavior of PG, we computed electronic structures and DOS for PG, h-PG, f-PG, and Cl-PG. Band structures along the symmetry line Γ−X−M−Γ are shown in Figure 2. As expected, h-PG and f-PG had indirect bandgaps of 4.97 eV and 4.81 eV, respectively. These values are larger than previously reported using the PBE functional to compute bandgaps [32,34,35,36,37,38]. These calculated bandgap values indicate that both hydrogenation and fluorination can tune the electronic structure of PG and change it from a semiconductor to an insulator. In contrast to these results, a metal–semiconductor transition was observed after chlorine functionalization. This result is interesting given previous theoretical observations of chlorine-functionalized graphene, where a direct bandgap of 2.81 eV was obtained using the HSE06 functional [39]. However, experimentally, a small bandgap of 0.045 eV was measured in a partially chlorinated graphene sample [40], suggesting that our numerical results, which showed a metallic behavior, may offer a pathway for studying and understanding Cl-PG. Variation in the energy gaps of PG, h-PG, f-PG, and Cl-GP is shown in Figure 3.

**Figure 4:**
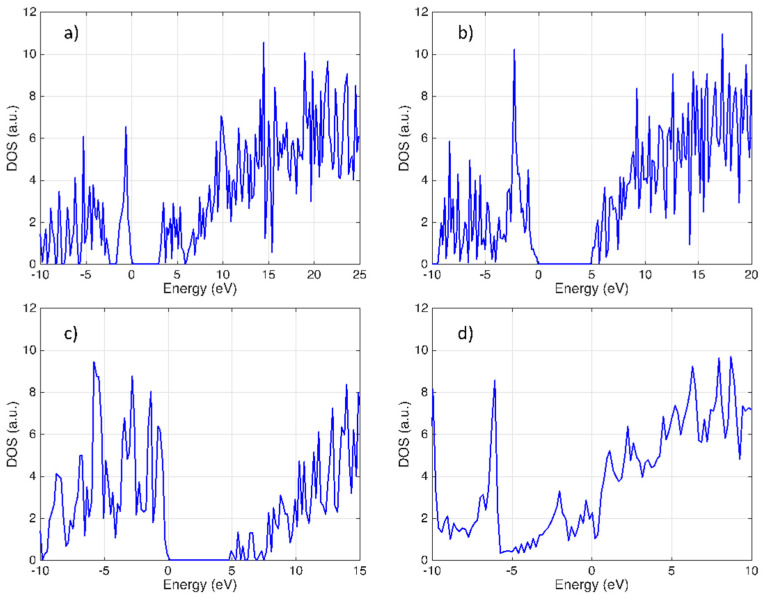
Density of states of (**a**) PG, (**b**) h-PG, (**c**) f-PG, and (**d**) Cl-PG.

Also, we calculated the DOS because it influences electronic, thermal, and optical properties, and to predict the bandgap of PG and functionalized variations more accurately. Figure 4 compares the DOS of these compounds; we can see a bandgap for PG, h-PG, and f-PG, but no bandgap for Cl-PG. The difference in the DOS between h-PG, f-PG, and Cl-PG can be attributed to the electronegativity of hydrogen, fluorine, and chlorine atoms.

### 3.3. Discussion

Although the synthesis of high-quality functionalized 2D materials remains a significant challenge, synthesis techniques already implemented for other 2D materials may provide a path to investigate the properties of PG and its functionalized forms experimentally. Graphene and transition metal oxides (TMOs) have been synthesized using selective extraction methods [41]. Moreover, chemical vapor deposition (CVD) methods for doping graphene [42] could be explored to functionalize PG. Regarding characterization, several customary techniques have been used. To extract nanostructure information, techniques such as scanning electron microscopy (SEM), transmission electron microscopy (TEM), or atomic force microscopy (AFM) are employed. Furthermore, the chemical composition of 2D materials has been studied by thermogravimetric analysis (TGA), X-ray photoelectron spectroscopy (XPS), and infrared spectroscopy (IT). Additionally, electronic structure can be studied by photoluminescence (PL) spectroscopy and ultraviolet photoelectron spectroscopy (UPS) [43]. Due to their novel properties and their ability to be tuned by functionalization, we foresee various applications relevant to nanoelectronics. For example, PG has lower thermal conductivity than graphene, making it more suitable for improving the thermoelectric figure of merit [44]. Also, functionalized forms of PG could be incorporated as an absorption element into multi-junction solar cell devices due to their modified bandgaps. In the same context of nanoelectronics, the materials studied could be implemented as transparent electrodes or hole/electron transport materials.

## 4. Conclusions

In this study, we investigated the effect of functionalization on the electronic properties of PG using first-principles DFT calculations. We studied four configurations: penta-graphene (PG), hydrogenated penta-graphene (h-PG), fluorinated penta-graphene (f-PG), and chlorinated penta-graphene (Cl-PG). Our calculations show that the indirect bandgaps of PG, h-PG, and f-PG are 3.05 eV, 4.97 eV, and 4.81 eV, respectively, showing that both hydrogenation and fluorination can effectively tune the electronic structure of PG, transforming it from a semiconductor to an insulator. In contrast, the Cl-PG bandgap closes, indicating a metallic behavior. These results suggest that functionalization can effectively tune the electronic properties of PG, which may provide useful guidance for incorporating novel materials in nanoelectronics devices. Future studies and modifications such as doping, chemical modification, electrostatic control, and alloying can mitigate material shortcomings, thereby enhancing PG’s advantages and facilitating the realization of 2D material-based optoelectronics.

## Figures and Tables

**Table 1:** Computational details.

Computational Details	
Software	VASP (Version: VASP.6.4.0)
Exchange-correlation functional	GGA-PBE
Pseudopotentials	Projector augmented wave (PAW)-PBE
Smearing	Gaussian smearing
C-valence configuration	2s^2^2p^2^, 4 electron valence number, 147.1560 eV
H-valence configuration	1s^1^, 1 electron valence number, 12.4884 eV
F-valence configuration	2s^2^2p^5^, 7 electron valence number, 659.4942 eV
Cl-valence configuration	3s^2^3p^5^, 12 electron valence number, 409.7259 eV
Plane wave basis set cut-off energy	500 eV
Vacuum space in the z-direction	15 Å
k-point mesh	8 × 8 × 1 Monkhorst–Pack
Electronic band calculation	25 × 25 × 1

**Table 2:** Literature values and our optimized lattice parameters for all PGs and functionalized PGs studied.

Compound	Literature Values		Our Optimized Values	
	Parameters (Å)		Parameters (Å)	
PG	a	3.64	a	3.63
h-PG	a	3.4897	a	3.50
f-PG	a	3.4897	a	3.55
Cl-PG	a	3.4897	a	3.88

Sources of literature values: PG [32,33,34,35,36], h-PG [32,33,34,35,36,37], f-PG [32,33,34,35,36,37], and Cl-PG [32].

**Table 3:** Our optimized structured parameters, distance (*d*) of bonds, angle (θ), and height (h) between the carbon atoms’ top and bottom layers, and electronic bandgap calculations for PG and functionalized PG.

Parameters	Our Optimized Parameters			
	PG	h-PG	f-PG	Cl-PG
*d* (C–C) (Å)	1.549	1.098	1.357	1.581
*d* (C_1_–C_2_) (Å)	—	1.551	1.561	1.668
*d* (C_2_–C_2_) (Å)	—	1.55	1.559	1.663
$\theta_{C_{1}–C_{2}–C_{1}}$	—	105.9°	107.2°	114.6°
$\theta_{C_{2}–C_{1}–C_{2}}$	—	116.9°	119.5°	129.7°
h (Å)	1.21	1.62	1.61	1.55
Bandgap (HSE06) (eV)	3.05	4.97	4.81	—

## Data Availability

The original contributions presented in the study are included in the article, further inquiries can be directed to the corresponding author.
